# Investigation of thermal performance of Maxwell hybrid nanofluid boundary value problem in vertical porous surface via finite element approach

**DOI:** 10.1038/s41598-022-06213-8

**Published:** 2022-02-11

**Authors:** Ebrahem A. Algehyne, Essam R. El-Zahar, S. H. Elhag, Fatimah S. Bayones, Umar Nazir, Muhammad Sohail, Poom Kumam

**Affiliations:** 1 Department of Mathematics, Faculty of Science, University of Tabuk, P.O. Box 741, Tabuk, 71491 Saudi Arabia; 2 Nanotechnology Research Unit (NRU), University of Tabuk, Tabuk, 71491 Saudi Arabia; 3 Department of Mathematics, College of Science and Humanities in Al-Kharj, Prince Sattam Bin Abdulaziz University, P.O. Box 83, Al-Kharj, 11942 Saudi Arabia; 4 Department of Basic Engineering Science, Faculty of Engineering, Menoufia University, Shebin El-Kom, 32511 Egypt; 5 Department of Mathematics and Statistics, College of Science, Taif University, P.O. Box 11099, Taif, 21944 Saudi Arabia; 6 Department of Applied Mathematics and Statistics, Institute of Space Technology, P.O. Box 2750, Islamabad, 44000 Pakistan; 7 Center of Excellence in Theoretical and Computational Science (TaCS-CoE) & KMUTT Fixed Point Research Laboratory, Room SCL 802 Fixed Point Laboratory, Science Laboratory Building, Department of Mathematics, Faculty of Science, King Mongkut’s University of Technology Thonburi (KMUTT), 126 Pracha-Uthit Road, Bang Mod, Thung Khru, Bangkok, 10140 Thailand; 8 Department of Medical Research, China Medical University Hospital, China Medical University, Taichung, 40402 Taiwan

**Keywords:** Mathematics and computing, Nanoscience and technology

## Abstract

The study of thermo-physical characteristics is essential to observe the impact of several influential parameters on temperature and velocity fields. The transportation of heat in fluid flows and thermal instability/stability is a charming area of research due to their wider applications and physical significance because of their utilization in different engineering systems. This report is prepared to study thermal transportation in Maxwell hybrid nanofluid past over an infinite stretchable vertical porous sheet. An inclusion of hybrid nanofluid is performed to monitor the aspects of thermal transportation. Keeping in mind the advantages of thermal failure, non-Fourier theory for heat flux model is utilized. Aspects of external heat source are also considered. The mathematical formulation for the considered model with certain important physical aspects results in the form of coupled nonlinear PDEs system. The obtained system is reduced by engaging boundary layer approximation. Afterwards, transformations have been utilized to convert the modeled PDEs system into ODEs system. The converted nonlinear ODEs system is then handled via finite element method coded in symbolic computational package MAPLE 18.0. Grid independent survey is presented for the validation of used approach and the comparative analysis has been done to confirm the reliability of obtained solution. The obtained solution is discussed and physical aspects have been explored and recorded against numerous involved influential variables. Motion into hybrid nanoparticles and nanoparticles becomes slow down versus higher values of Forchheimer and Darcy’s porous numbers. Thermal growth is enhanced for the case of hybrid nano-structures rather than for case of nanofluid. Thickness regarding momentum layer is dominated for hybrid nanoparticles rather than case of nanoparticles.

## Introduction

Modeling of complex phenomenon to understand the mathematical physics behind them is the growing and useful tool for researchers, mathematicians and engineers. Several important empirical relations have conducted and proposed to model the nature of complex phenomenon arising in mathematical physics.

Several important contributions have been performed so far on Maxwell model. For instance, Mushtaq et al.^1^ examined the buoyancy force effects by considering generalized heat flux theory under variable and constant wall temperature past over an elastic vertical plate. They examined the impact on opposing and assisting flow for different emerging parameters. They recorded the increase in temperature field against buoyancy parameter and decrease in velocity field against fluid relaxation time. Zhang et al.^2^ computed exact solution of unsteady fractional Maxwell model and thermal transport mechanism is highlighted via Laplace transform procedure. They have plotted several graphs and analyzed the contribution of numerous emerging parameters in the presence/absence of slip factor. They have shown the decline in temperature profile against fractional parameters and upsurges is monitored in velocity against the said parameters. Numerical approach coupled with Lie group investigation has been applied by Shafiq et al.^3^ to examine the flow of Maxwell model past over a stretched penetrable sheet. They noticed the opposite behavior in thermal profile for heat absorption/generation parameter. Moreover, they noticed the increase in fluid velocity for Deborah number. Raza and Ullah^4^ used two different fractional derivatives approaches to study thermal transportation in unsteady Maxwell model. They computed the solution via Laplace procedure. They examined the increase in fluid velocity against Maxwell fluid parameter and Grashof number. Kaushik et al.^5^ examined the flow of Maxwell model in rotating channel. They obtained streamlines for velocity solution and impact of numerous parameters on solution is explored and explained in detail. Thermal transportation in MHD fractional Maxwell model with dissipation was studied by Bai et al.^6^. They used numerical and analytical approach to discuss the solution behavior against numerous involved parameters. They monitored the decline in velocity for magnetic parameter and similar bearing of Prandtl number in thermal field. Iqbal et al.^7^ worked on convective Maxwell model obeying peristaltic transport phenomenon in a channel. Transportation of heat and mass is also investigated by them. They recorded the trapping phenomenon. They examined the depreciation in concentration against Schmidt and Soret numbers. Furthermore, opposite contribution of Biot number is monitored for concentration and temperature profiles.

Extensive utilization of nanoparticles^8–10^ in different mechanisms including medicines, cancer treatment, machineries and different other household instruments make a remarkable attention of this topic. Growing researches and development in technology accept the involvement and significance of nanoparticles. Several researchers pay their attention and contributed on the topic. For instance, Abdelsalam and Sohail^11^ studied bi-directional bio-convective stretched flow with variable thermophysical attributes. They obtained the resulting reduced PDEs after utilizing boundary layer theory. They solved the converted ODEs from PDEs with the addition of suitable transformation by OHAM tool. They established that higher order approximations reduce the error. They recorded the depreciation in velocity field against magnetic parameter and upsurges in temperature profile. Furthermore, decline in concentration profile is observed against Schmidt number. Mixing of graphene nanoparticles in Eyring Powell MHD unsteady model with momentum slip past over a stretched sheet was examined by Khan et al.^12^. Ghadikolaei et al.^13^ worked on magneto nanofluid Casson model in the presence of inclined non-uniform magnetic field. They considered radiation, viscous dissipation and Joule heating in temperature expression. Shooting approach has been utilized for the solution of nonlinear coupled ODEs. They recorded the augmentation in temperature field against Casson parameter and decline in fluid velocity. Ramzan et al.^14^ addressed the mixed convection phenomenon in stretched nanofluid model of viscoelastic material with Soret and Dufour effects. They monitored the resistance in thermal profile against mounting values of Prandtl number. Rotating MHD CNTs mixed Casson fluid flow under radiation and heat generation effects was studied by Muhammad et al.^15^. Hady et al.^16^ studied saturated yield exhibiting flow past over a porous surface. They treated nonlinear modeled equations numerically. They recorded the depreciation in Nusselt and Sherwood numbers against yield stress parameter. Nayak et al.^17^ studied stretched MHD radiated viscous fluid over an exponential stretching sheet numerically via shooting method. They have examined the involvement of different nanoparticles and monitored the heat transportation rate. Aman et al.^18^ examined the inclusion of different nanoparticles mixture in Maxwell model. Additionally, the involvement is SWCNTs and MWCNTs have been presented to notice thermal performance. They handled the resulting nonlinear equations analytically. Few important latest contributions containing several important aspects are covered in Refs.^19–21^.

The manufacturing of hybrid nanoparticles has been developed by scattering nanofluids while hybrid nanofluid can boost ability of thermal properties in nanofluids. Main role of hybrid nanofluid has been improved thermal conductivity, low cost and stability. Such role of hybrid nanofluid makes it favorite in field of nanotechnology. Therefore, hybrid nanofluid has been produced great interest for researchers. Some study related to hybrid nanofluid is mentioned here. For example, Hanif et al.^22^ considered heated cone to investigate thermal features under magnetic field imposing hybrid nanoparticles whereas they have estimated entropy generation. Khan et al.^23^ studied thermal aspects using silicon dioxide and molybdenum disulfide over a 3D heated surface. Hanif et al.^24^ studied performance of hybrid nanofluid under thermal radiation in heated cone in the presence of magnetic field numerical solved by Crank-Nicolson approach. Hanif et al.^25^ scrutinized a novel investigation of viscosity model for unsteady flow using magnetic field imposing hybrid nanofluid. Saqib et al.^26^ discussed heat energy characterizations in the presence of hybrid nanoparticles in form of fractional DEs (differential equations) using integral transforms. Saqib et al.^27^ analyzed energy transfer under MHD flow in rheology of Maxwell liquid in view of Cattaneo–Friedrich model. Hanif et al.^28^ investigated magneto-hydrodynamic characterizations in view of nanoparticles towards a vertical porous heated cone. Jamil et al.^29^ investigated features of Maxwell martial considering effect of chemical reaction in form fractional derivatives using thermal radiation. Anwar et al.^30^ studied microscopic investigation under magnetic field of inertial motion considering porous geometry. Dinarvand et al.^31^ discussed improvement in micro-circulatory process inserting role of hybrid nano-structures over a porous surface. Mousavi et al.^32^ investigated performance of Casson liquid and dual solutions of developed model using nanoparticles over an expanding surface. Dinarvand et al.^33^ analyzed swirling flow in mass-based model using hybrid nanoparticles via von Kármán’s theory. Aghamajidi et al.^34^ discussed MHD motion and connective heat transfer using Tiwari-Das model related to nanoparticles over a cone. Dinarvand et al.^35^ developed boundary layer model inserting hybrid nano-structures. Dinarvand et al.^36^ analyzed an effect of magnetic field using MgO–Ag nanoparticles over moving heated needle.

Two-dimensional model related to heat transfer for unsteady flow is developed past a vertical surface. A phenomenon for hybrid nanoparticles is imposed in the presence of non-Fourier’s theory for unsteady flow along with heat generation term. A rheology of Maxwell martial is studied with Darcy’s Forchheimer theory using various thermal properties. A finite element method is implemented to simulate numerical results. It is noticed that such developing model is not investigated yet. The conducted research is organized as: literature survey is included in “Introduction” section, modeling is covered in “Physical statement” section with important physical quantities, an effective and convergent as well as stable numerical approach is presented in “Procedure of finite element method” section, results with physical interpretation are mentioned in “Results and discussion” section and consequences related considered problem have been reported in “Consequences of current problem” section.

## Physical statement

**Figure 1:**
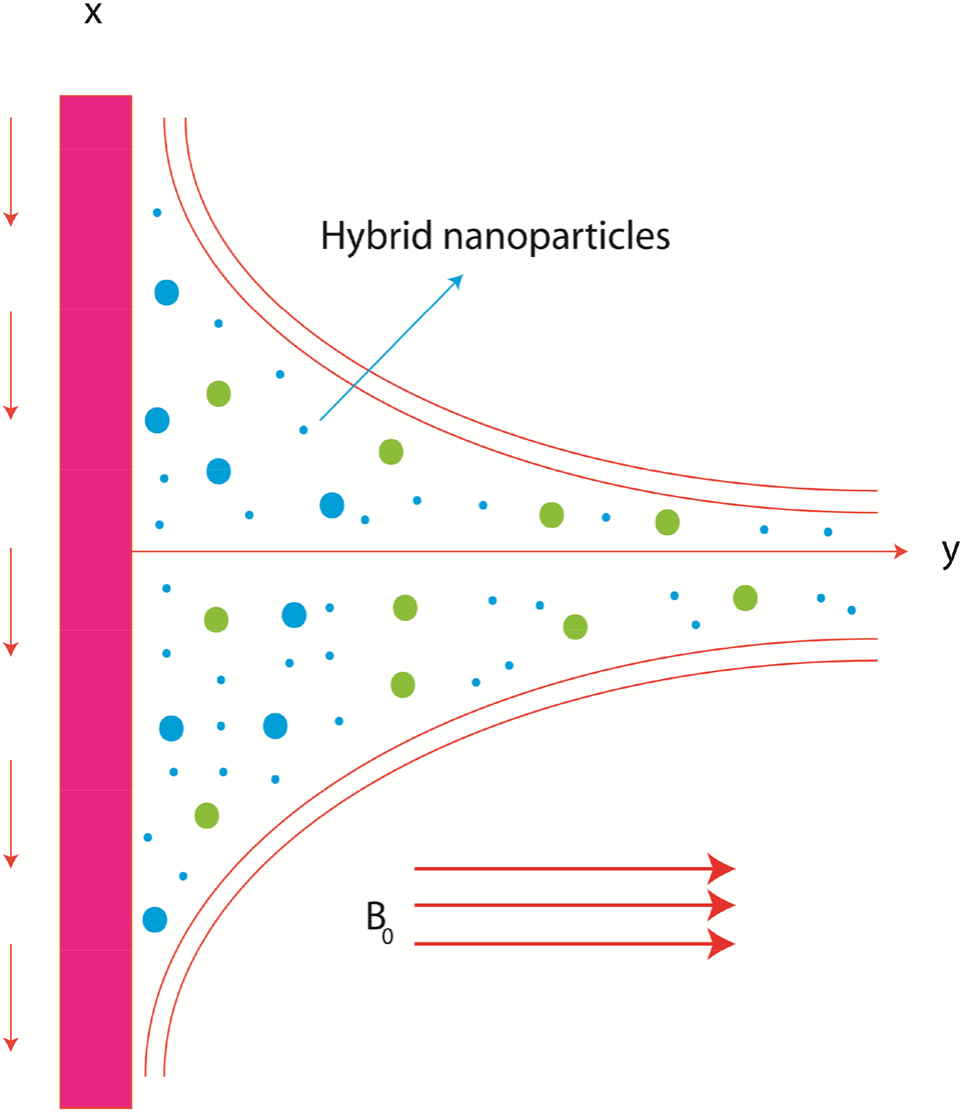
Geometry of current model.

Characteristics of thermal energy in Maxwell liquid inserting dispersion of nanomaterials and hybrid nanomaterials are considered past melting vertical surface as shown in Fig. 1. Phenomena of heat energy characteristics are occurred due to heat generation and magnetic field. Mechanism of non-Fourier’s law is modeled in energy equation. Theory of Darcy’s Forchheimer is observed. The fluid runs over melting surface because of movement of wall. The behavior of fluid rheology is captured by Fig. 1. It is noticed that movement of wall is occurred along y-direction and constant magnetic field is assumed along normal direction. Amount of nanoparticles and hybrid nano-structures is considered above x-direction. A vertical surface is considered to analyze feature of considered effects. Governing physical system of equations contains the utilization of following:

- two dimensional unsteady stretching porous sheet;
- mixed convective flow;
- single phase model;
- hybrid nanoparticles;
- viscoelastic material (Maxwell model);
- heat generation/absorption;
- non-Fourier heat flux;
- no slip theory;
- boundary layer analysis.

The PDEs are modeled trough concept of boundary layer for the conservation laws and modeled PDEs^38^ are

1 $$0 = \frac{\partial v}{{\partial y}} + \frac{\partial u}{{\partial x}},$$

2 $$\tfrac{\partial u}{{\partial t}} + v\tfrac{\partial u}{{\partial y}} + u\tfrac{\partial u}{{\partial x}} + \lambda_{1} \left( {\tfrac{{\partial^{2} u}}{{\partial t^{2} }} + u^{2} \tfrac{{\partial^{2} u}}{{\partial x^{2} }} + v^{2} \tfrac{{\partial^{2} u}}{{\partial y^{2} }} + 2uv\tfrac{\partial u}{{\partial x\partial y}}} \right) = \nu_{hnf} \tfrac{{\partial^{2} u}}{{\partial y^{2} }} - \tfrac{{\nu_{hnf} }}{{k^{ * } }}F_{s} u - \tfrac{{F_{s} }}{{(k^{ * } )^{1/2} }}u^{2} + G\beta_{hnf} (T - T_{\infty } ),$$

3 $$\tfrac{\partial T}{{\partial t}} + u\tfrac{\partial T}{{\partial x}} + v\tfrac{\partial T}{{\partial y}} + \lambda_{2} \left( {\begin{array}{*{20}c} {\tfrac{{\partial^{2} T}}{{\partial t^{2} }} + u\tfrac{\partial u}{{\partial x}}\tfrac{\partial T}{{\partial x}} + v\tfrac{\partial v}{{\partial y}}\tfrac{\partial T}{{\partial y}} + u\tfrac{\partial v}{{\partial x}}\tfrac{\partial T}{{\partial y}} + v\tfrac{\partial u}{{\partial y}}\tfrac{\partial T}{{\partial x}} + 2uv\tfrac{{\partial^{2} T}}{\partial x\partial y}} \\ {\tfrac{\partial u}{{\partial t}}\tfrac{\partial T}{{\partial x}} + u^{2} \tfrac{{\partial^{2} T}}{{\partial x^{2} }} + v^{2} \tfrac{{\partial^{2} T}}{{\partial y^{2} }} - \tfrac{{Q_{0} }}{{(\rho C_{p} )_{hnf} }}(u\tfrac{\partial T}{{\partial x}} + v\tfrac{\partial T}{{\partial y}}) + 2u\tfrac{{\partial^{2} T}}{\partial x\partial t}} \\ {\tfrac{\partial v}{{\partial t}}\tfrac{\partial T}{{\partial y}} + 2v\tfrac{{\partial^{2} T}}{\partial y\partial t} - Q_{0} \tfrac{\partial T}{{\partial t}}} \\ \end{array} } \right) = \tfrac{{K_{hnf} }}{{(\rho C_{p} )_{hnf} }}\tfrac{{\partial^{2} T}}{{\partial^{2} y}} + \tfrac{{Q_{0} }}{{(\rho C_{p} )_{hnf} }}(T - T_{\infty } ),$$

where velocity is $[u,\,\;v,\,\;0],\,\;\lambda_{1} \;$ is the relaxation time,$\;F_{s} \;$ is the inertia coefficient, permeability is denoted by $k^{ * } ,\,\;T\;$ is the temperature, thermal relaxation time is $\lambda_{2} ,\,\;$ heat generation is $Q_{0} ,\,\;$ temperature in ambient fluid is $T_{\infty } ,\,\;K\;$ is the thermal conductivity, fluid density is represented by $\rho ,\,$ specific heat is $C_{p} \;$ and $hnf$ stands hybrid nanofluid, gravitational acceleration is $\rho$ and $\beta_{hnf}$ is thermal expansion coefficient.

Boundary conditions regarding problem are

4 $$\left. {\begin{array}{*{20}l} {v = u = 0,\,\;T_{w} \; = T\,{\text{when }}t < 0} \hfill \\ {u = \tfrac{ax}{{1 - ct}},\,\;v = 0,\,\;T = T_{w} \;{\text{at}}\;y = 0\;{\text{and}}\;t \ge 0} \hfill \\ {u = 0,\,\;T = T_{\infty } ,\,\;{\text{at}}\;y = \infty \;{\text{and}}\;t \ge 0} \hfill \\ \end{array} } \right\}.$$

Here, $a$ and $c$ are constant number whereas $a$ and $c$ have $s^{ - 1}$ unit. The relationships among thermo-physical properties of pure fluid, mono, hybrid nanoparticles and mixture fluid are mentioned^21,38^ below and their values are mentioned in Table 1.

5 $$\left. {\begin{array}{*{20}c} {\rho_{nf} = (1 - \varphi )\rho_{f} + \varphi \rho_{s} ,\,\;(\rho c_{p} )_{nf} = (1 - \varphi )(\rho c)_{f} + \varphi (\rho c)_{s} } \\ {\mu_{nf} = \tfrac{{\mu_{f} }}{{(1 - \varphi )^{{\tfrac{5}{2}}} }},\,\;k_{nf} = \left\{ \tfrac{{k_{s} + 2k_{f} - 2\varphi (k_{f} - k_{s} )}}{{k_{s} + 2k_{f} + \varphi (k_{f} - k_{s} )}}\right\} ,\,\;\sigma_{nf} = (1 + \tfrac{3(\sigma - 1)\varphi }{{\sigma + 2 - (\sigma - 1)\varphi }})} \\ \end{array} } \right\}\,,$$

6 $$\left. {\begin{array}{*{20}l} {\rho_{hnf} = \left[ {(1 - \varphi_{2} )\{ (1 - \varphi_{1} )\rho_{f} + \varphi_{1} (\rho c_{p} )_{{s_{1} }} \} } \right] + \varphi_{2} (\rho c_{p} )_{{s_{2} }} } \hfill \\ {\rho c_{p} )_{hnf} = [(1 - \varphi_{2} )\{ (1 - \varphi_{1} )(\rho c_{p} )_{f} + \varphi_{1} (\rho c_{p} )_{{s_{1} }} \} ] + \varphi_{2} (\rho c_{p} )_{{s_{2} }} } \hfill \\ {\mu_{hnf} = \tfrac{{\mu_{f} }}{{(1 - \varphi_{1} )^{2.5} (1 - \varphi_{2} )^{2.5} }},\,\;\tfrac{{K_{hnf} }}{{K_{f} }} = \tfrac{{k_{{s_{2} }} + (n - 1)k_{bf} - (n - 1)\varphi_{2} (k_{bf} - k_{{s_{2} }} )}}{{k_{{s_{2} }} + (n - 1)k_{bf} + \varphi_{2} (k_{bf} - k_{{s_{2} }} )}}} \hfill \\ \begin{gathered} \tfrac{{K_{bf} }}{{K_{f} }} = \tfrac{{k_{{s_{1} }} + (n - 1)k_{f} - (n - 1)\varphi_{1} (k_{f} - k_{{s_{1} }} )}}{{k_{{s_{1} }} + (n - 1)k_{f} + \varphi_{1} (k_{f} - k_{{s_{1} }} )}},\,\;\tfrac{{\sigma_{bf} }}{{\sigma_{f} }} = \tfrac{{\sigma_{{s_{1} }} + 2\sigma_{f} - 2\varphi_{1} (\sigma_{f} - \sigma_{{s_{1} }} )}}{{\sigma_{{s_{1} }} + 2\sigma_{f} + \varphi_{1} (\sigma_{f} - \sigma_{{s_{1} }} )}} \hfill \\ \tfrac{{\sigma_{nf} }}{{\sigma_{f} }} = \left( {1 + \tfrac{3(\sigma - 1)\phi }{{(\sigma + 2) - (\sigma - 1)\phi }}} \right),\,\tfrac{{\sigma_{hnf} }}{{\sigma_{bf} }} = \tfrac{{\sigma_{{S_{2} }} + 2\sigma_{bf} - 2\phi_{2} (\sigma_{bf} - \sigma_{{S_{2} }} )}}{{\sigma_{{S_{2} }} + 2\sigma_{bf} + \phi_{2} (\sigma_{bf} - \sigma_{{S_{2} }} )}} \hfill \\ \end{gathered} \hfill \\ \end{array} } \right\}.$$

**Table 1:** Thermal properties^37,38^ for silver, ethylene glycol and molybdenum disulfide.

$MoS_{2}$	$Ag$	EG
$\rho_{{MoS_{2} }} = 5060$	$\rho_{Ag} = 10480$	$\rho_{EG} = 1113.5$
$(C_{p} )_{{MoS_{2} }} = 397.21$	$(C_{p} )_{{_{Ag} }} = 235$	$(C_{p} )_{{_{EG} }} = 2430$
$k_{{MoS_{2} }} = 904.4$	$k_{{_{Ag} }} = 429$	$k_{{_{EG} }} = 0.253$
$\sigma_{{MoS_{2} }} = 2.09 \times 10^{ - 5}$	$\sigma_{{_{Ag} }} = 6.30 \times 10^{7}$	$\sigma_{{_{EG} }} = 4.3 \times 10^{ - 5}$

Following change of variables is suggested for non-dimensional PDEs

7 $$\left. {\begin{array}{*{20}c} {u = ax(1 - ct)^{ - 1} f^{\prime},\,\;v = - (\tfrac{{a\nu_{f} }}{1 - ct})^{1/2} f} \\ {\eta = \left( {\tfrac{a}{{\nu_{f} }}} \right)^{1/2} (1 - ct)^{ - 1/2} y,\,\;\theta = \tfrac{{T - T_{\infty } }}{{T_{w} - T_{\infty } }}} \\ \end{array} } \right\},$$

8 $$\left. {\begin{array}{*{20}l} {f^{\prime\prime\prime} - \varepsilon f^{\prime} - \tfrac{{\nu_{f} }}{{\nu_{hnf} }}\left[ {f^{{\prime^{2} }} - ff^{\prime\prime} + \lambda^{ * } (f^{\prime} - \tfrac{1}{2}\eta f^{\prime\prime})} \right] - \tfrac{{\nu_{f} }}{{\nu_{hnf} }}\gamma \left( {\begin{array}{*{20}c} {f^{2} f^{\prime\prime} - 2ff^{\prime}f^{\prime\prime} + \lambda^{ * } (f^{\prime} + } \\ {\eta f^{\prime\prime} + \eta^{2} f^{\prime\prime\prime})} \\ \end{array} } \right)} \hfill \\ { - \tfrac{{\nu_{f} }}{{\nu_{hnf} }}Frf^{{\prime^{2} }} + \lambda \theta = 0,} \hfill \\ {f(0) = 0,\,\;f^{\prime}(0) = 1,\,\;f(\infty ) = 0} \hfill \\ \end{array} } \right\},$$

9 $$\left. {\begin{array}{*{20}l} {\theta^{\prime\prime} + \tfrac{{K_{f} (\rho C_{p} )_{hnf} }}{{K_{hnf} (\rho C_{p} )_{f} }}\Pr \left\{ {f\theta^{\prime} - \lambda^{ * } \tfrac{1}{2}\eta \theta^{\prime}} \right\} + \tfrac{{K_{f} (\rho C_{p} )_{hnf} }}{{K_{hnf} (\rho C_{p} )_{f} }}\delta \Pr (f^{2} \theta^{\prime\prime} + ff^{\prime}\theta^{\prime} + \lambda^{ * } f\theta^{\prime})} \hfill \\ {\tfrac{1}{2}\tfrac{{K_{f} (\rho C_{p} )_{hnf} }}{{K_{hnf} (\rho C_{p} )_{f} }}\Pr \delta \lambda^{ * } (f\theta^{\prime} + \eta f^{\prime}\theta^{\prime}) - \tfrac{1}{2}\tfrac{{K_{f} (\rho C_{p} )_{hnf} }}{{K_{hnf} }}\delta \Pr \lambda^{ * } Hs\eta \theta^{\prime} + } \hfill \\ { - \tfrac{1}{4}\tfrac{{K_{f} (\rho C_{p} )_{hnf} }}{{K_{hnf} (\rho C_{p} )_{f} }}\Pr \delta \lambda^{{^{2} * }} (3\eta \theta^{\prime} + \eta^{2} \theta^{\prime\prime}) + \delta \tfrac{{K_{f} }}{{K_{hnf} }}\Pr Hsf\theta^{\prime} + \tfrac{{K_{f} }}{{K_{hnf} }}Hs\Pr \theta = 0} \hfill \\ {\theta (0) = 1,\,\;\theta (\infty ) = 0} \hfill \\ \end{array} } \right\}.$$

The porosity, Deborah, Forchheimer, Prandtl, heat generation, thermal relaxation and unsteadiness numbers are captured below

10 $$\varepsilon = \frac{{F_{s} \nu_{f} }}{{k^{ * } a(1 - ct)}},\,\;\gamma = \lambda_{1} a,\,\;Fr = = \frac{{xF_{s} }}{{\sqrt {k^{ * } } }},\,\;\mathop {\Pr }\limits = \frac{{\mu_{f} (C_{p} )_{f} }}{{K_{f} }},\,\;$$

11 $$\delta = \frac{{a\lambda_{2} }}{1 - ct},\,\;\lambda^{ * } = \frac{c}{a},\,\;Hs = \frac{{Q_{0} (1 - ct)}}{{(C_{p} )_{f} a\rho_{f} }}.$$

Coefficient associated with drag force is

12 $$C_{f} = \frac{{\left. {\tau_{xy} } \right|_{y = 0} }}{{\rho_{hnf} (u_{w} )^{2} }},\,\;(Re)^{1/2} C_{f} = \frac{1}{{(1 - \varphi_{1} )^{2.5} (1 - \varphi_{2} )^{2.5} }}f^{\prime\prime}(0).$$

Rate of heat energy is

13 $$Nu = \frac{{xq_{w} }}{{K_{f} (T_{w} - T_{\infty } )}},\,\;q_{w} = - K_{hnf} \frac{\partial T}{{\partial y}}|_{y = 0} ,\,\;(Re)^{1/2} Nu = - \frac{{K_{hnf} }}{{K_{f} }}\theta^{\prime}(0).$$

Reynolds parameter is illustrated by ${\text{Re}} \left( = \tfrac{{ax^{2} }}{{\nu_{f} (1 - ct)}}\right).$

## Procedure of finite element method

Finite element method is implemented and computer code is developed to handle the complex coupled (ordinary differential equations) ODEs which results after modeling the Maxwell fluid model with heat transport. The procedure consists of following key steps.

- Residuals of current ODEs are integrated and weighted and residuals of considered problem are
  14 $$\int \nolimits_{{\eta_{e} }}^{{\eta_{e + 1} }} WT_{1} [f^{\prime} - H]d\eta = 0,\,$$
  15 $$\int \nolimits_{{\eta_{e} }}^{{\eta_{e + 1} }} WT_{2} \left[ {H^{\prime\prime} - \varepsilon H - \tfrac{{\nu_{f} }}{{\nu_{hnf} }}(H^{2} - fH^{\prime} + \lambda^{ * } (H - \tfrac{1}{2}\eta H^{\prime})) - \tfrac{{\nu_{f} }}{{\nu_{hnf} }}FrH^{2} + \lambda \theta - \tfrac{{\nu_{f} }}{{\nu_{hnf} }}\gamma \left( {\begin{array}{*{20}c} {f^{2} H^{\prime} - 2fHH^{\prime} + \lambda^{ * } (H + } \\ {\eta H^{\prime} + \eta^{2} H^{\prime\prime})} \\ \end{array} } \right)} \right]d\eta = 0,\,$$
  16 $$\int \nolimits_{{\eta_{e} }}^{{\eta_{e + 1} }} WT_{3} \left[ {\theta^{\prime\prime} + \tfrac{{K_{f} (\rho C_{p} )_{hnf} }}{{K_{hnf} (\rho C_{p} )_{f} }}\Pr \left\{ {f\theta^{\prime} - \lambda^{ * } \tfrac{1}{2}\eta \theta^{\prime}} \right\} + \tfrac{{K_{f} (\rho C_{p} )_{hnf} }}{{K_{hnf} (\rho C_{p} )_{f} }}\delta \Pr (f^{2} \theta^{\prime\prime} + fH\theta^{\prime} + \lambda^{ * } f\theta^{\prime})\tfrac{1}{2}\tfrac{{K_{f} (\rho C_{p} )_{hnf} }}{{K_{hnf} (\rho C_{p} )_{f} }}\Pr \delta \lambda^{ * } (f\theta^{\prime} + \eta H\theta^{\prime}) - \tfrac{1}{2}\tfrac{{K_{f} (\rho C_{p} )_{hnf} }}{{K_{hnf} }}\delta \Pr \lambda^{ * } Hs\eta \theta^{\prime} - \tfrac{1}{4}\tfrac{{K_{f} (\rho C_{p} )_{hnf} }}{{K_{hnf} (\rho C_{p} )_{f} }}\Pr \delta \lambda^{{^{2} * }} (3\eta \theta^{\prime} + \eta^{2} \theta^{\prime\prime}) + \delta \tfrac{{K_{f} }}{{K_{hnf} }}\Pr Hsf\theta^{\prime} + \tfrac{{K_{f} }}{{K_{hnf} }}Hs\Pr \theta } \right]d\eta = 0.$$
  Here, $WT_{1}$, $WT_{2}$ and $WT_{3}$ are weight functions and unknown variables ($\theta$,$f$ and $H$) are formulated in the presence of shape function ($\psi_{j}$) as
  17 $$f = \mathop \sum \limits_{j = 1}^{2} f_{j} \psi_{j} ,\,\;\theta = \mathop \sum \limits_{j = 1}^{2} \theta_{j} \psi_{j} \,,\,H = \mathop \sum \limits_{j = 1}^{2} H_{j} \psi_{j} .$$
- The weak procedures are made via weighted residuals.
- Stiffness matrices are calculated by using Galerkin approximations (into weak procedures) whereas Stiffness matrices are also used in assembly approach. The stiffness elements are constructed as
  18 $$\;K_{ij}^{12} = - \int \nolimits_{{\eta_{e} }}^{{\eta_{e + 1} }} (\psi_{j} )\psi_{i} d\eta ,\,B_{i}^{1} = 0,\,K_{ij}^{11} = \int \nolimits_{{\eta_{e} }}^{{\eta_{e + 1} }} \left(\frac{{d\psi_{j} }}{d\eta }\right)\psi_{i} d\eta ,\,K_{ij}^{13} = 0,\,\;$$
  19 $$K_{ij}^{22} = \int \nolimits_{{\eta_{e} }}^{{\eta_{e + 1} }} \left[ {\begin{array}{*{20}c} { - \frac{{d\psi_{j} }}{d\eta }\frac{{d\psi_{i} }}{d\eta } - \varepsilon \psi_{i} \psi_{j} - \tfrac{{\nu_{f} }}{{\nu_{hnf} }}(\overline{H} \psi_{i} \psi_{j} - f\psi_{i} \frac{{d\psi_{i} }}{d\eta } + \lambda^{ * } \left(\psi_{i} \psi_{j} - \tfrac{1}{2}\eta \psi_{i} \frac{{d\psi_{i} }}{d\eta })\right)} \\ { - \tfrac{{\nu_{f} }}{{\nu_{hnf} }}Fr\overline{H} \psi_{i} \psi_{j} - \tfrac{{\nu_{f} }}{{\nu_{hnf} }}\gamma \left( {\begin{array}{*{20}c} {\overline{{f^{2} }} \psi_{i} \frac{{d\psi_{i} }}{d\eta } - 2\overline{f} (\overline{H} )\psi_{i} \frac{{d\psi_{i} }}{d\eta } + \lambda^{ * } (\psi_{i} \psi_{j} } \\ { + \eta \psi_{i} \frac{{d\psi_{i} }}{d\eta } - \eta^{2} \frac{{d\psi_{j} }}{d\eta }\frac{{d\psi_{i} }}{d\eta })} \\ \end{array} } \right)} \\ \end{array} } \right]\,d\eta ,\,$$
  20 $$K_{ij}^{23} = \int \nolimits_{{\eta_{e} }}^{{\eta_{e + 1} }} \left[ {\lambda \psi_{i} \psi_{j} } \right]d\eta ,\,B_{i}^{2} = 0,\,K_{ij}^{31} = 0,K_{ij}^{32} = 0,B_{i}^{3} = 0,$$
  21 $$K_{ij}^{33} = \int \nolimits_{{\eta_{e} }}^{{\eta_{e + 1} }} \left[ {\begin{array}{*{20}c} { - \frac{{d\psi_{j} }}{d\eta }\frac{{d\psi_{i} }}{d\eta } - \tfrac{{K_{f} (\rho C_{p} )_{hnf} }}{{K_{hnf} (\rho C_{p} )_{f} }}\Pr \left\{ {\overline{F} \frac{{d\psi_{i} }}{d\eta }\psi_{j} - \lambda^{ * } \tfrac{1}{2}\eta \frac{{d\psi_{i} }}{d\eta }\psi_{j} } \right\}} \\ \begin{gathered} + \tfrac{{K_{f} (\rho C_{p} )_{hnf} }}{{K_{hnf} (\rho C_{p} )_{f} }}\delta \Pr ( - \overline{{f^{2} }} \frac{{d\psi_{j} }}{d\eta }\frac{{d\psi_{i} }}{d\eta } + f\overline{H} \frac{{d\psi_{i} }}{d\eta }\psi_{j} + \lambda^{ * } \overline{f} \frac{{d\psi_{i} }}{d\eta }\psi_{j} ) + \tfrac{{K_{f} }}{{K_{hnf} }}Hs\Pr \psi_{j} \psi_{i} \hfill \\ \tfrac{1}{2}\tfrac{{K_{f} (\rho C_{p} )_{hnf} }}{{K_{hnf} (\rho C_{p} )_{f} }}\Pr \delta \lambda^{ * } (\overline{f} \frac{{d\psi_{i} }}{d\eta }\psi_{j} + \eta \overline{H} \frac{{d\psi_{i} }}{d\eta }\psi_{j} ) - \tfrac{1}{2}\tfrac{{K_{f} (\rho C_{p} )_{hnf} }}{{K_{hnf} }}\delta \Pr \lambda^{ * } Hs\eta \frac{{d\psi_{i} }}{d\eta }\psi_{j} \hfill \\ - \tfrac{1}{4}\tfrac{{K_{f} (\rho C_{p} )_{hnf} }}{{K_{hnf} (\rho C_{p} )_{f} }}\Pr \delta \lambda^{{^{2} * }} (3\eta \frac{{d\psi_{i} }}{d\eta }\psi_{j} - \eta^{2} \frac{{d\psi_{j} }}{d\eta }\frac{{d\psi_{i} }}{d\eta }) + \delta \tfrac{{K_{f} }}{{K_{hnf} }}\Pr Hs\overline{f} \frac{{d\psi_{i} }}{d\eta }\psi_{j} \hfill \\ \end{gathered} \\ \end{array} } \right]\,d\eta .$$
- System of algebraic equations (nonlinear) is obtained.
- The algebraic system is linearized and solved iteratively. Convergence and mesh analysis are done and flow fields are recorded under the variation of physical parameters raised as a result of dimensional analysis. The error analysis of developed model is
  22 $$E_{rr} = \left| {\tau^{j} - \tau^{j - 1} } \right|\,\;{\text{and}}\;Maximum\left| {\tau^{j} - \tau^{j - 1} } \right| < 10^{ - 8} .$$
- Mesh free study is carried out and convergence is ensured.
- Mesh free analysis is displayed in Table 2 considering $\eta_{\max }$ is 8. It is observed that investigation related to mesh-free for 300 elements is simulated. Elements of desired domain are increased 30 elements to 300 elements whereas solution is converged for 300 elements and outcomes are not changed (affected) after 300 elements. It is concluded that solution is converged and solution becomes repeatable at 300 elements. Therefore, computation investigation is simulated within 300 elements of considered problem.

**Table 2:** Simulations for mesh-free in temperature and velocity observing 300 elements^38^ when $\varepsilon = 0.3,\,\lambda^{ * } = 0.7,\,\gamma = 1.5,\,Fr = 0.53,\,\Pr = 204,\;Hs = 1.7,\,\lambda = 1.3,\,\,\delta = 2.0,\,\varphi_{1} = 0.003,\,\varphi_{1} = 0.075.$

Number of elements	$f^{\prime}\left( {\frac{{\eta_{\max } }}{2}} \right)$	$\theta \left( {\frac{{\eta_{\max } }}{2}} \right)$
30	0.0075101	0.53284
60	0.0070855	0.51706
90	0.0070129	0.50973
120	0.0069580	0.50590
150	0.0066293	0.50517
180	0.0069777	0.50629
210	0.0065570	0.50628
240	0.0069647	0.50196
270	0.0071784	0.50850
300	0.0071764	0.50858

### Stopping condition and tolerance

Numerical simulations related to temperature gradient and skin friction coefficient are captured against various indicated numerical values of parameters. An indigenous computer is running and simulate considered problem in view of iterative manner. It is noticed that exact solution of considered problem is not available. Therefore, stooping condition is illustrated by an error $\left| {\tau^{j} - \tau^{j - 1} } \right| < \xi$. Here, $\xi$ is very small which is $10^{ - 8}$ and $\tau^{j}$ is almost equal to $\tau^{j - 1}$. Numerical values of Nusselt number and skin friction coefficient are noted when above condition is satisfied. Errors related various parameters are also simulated by Table 4.

## Results and discussion

The constructing model including various characteristics is observed past a heated vertical porous plate. A hybrid approach along with non-Fourier’s theory inserting heat generation number provides complex model. Such complex model is solved by finite element method. It is mentioned that mixture of $MoS_{2}$ and $Ag$ is known as hybrid nanofluid and $Ag$ is investigated as a nanofluid. Graphs related velocity and thermal energy are plotted among comparison nanofluid and hybrid nanoparticles versus physical parameters. The graphical impacts are given below.

### Visualization of flow characteristics via change in physical parameters

**Figure 2:**
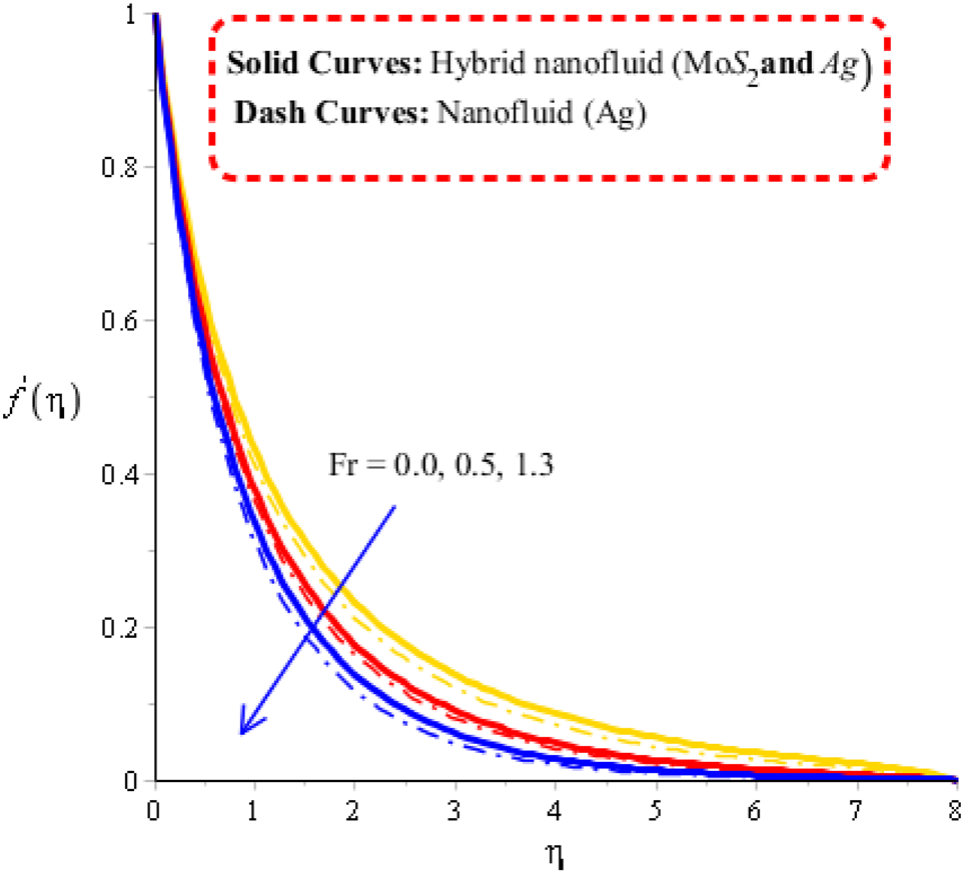
Velocity curves against $Fr$ when $\varepsilon = 0.23,\,\lambda^{ * } = 0.2,\,\gamma = 1.3,\,\,\Pr = 204,Hs = - 1.3,\,\lambda = 1.2,\,\delta = 2.0,\varphi_{1} = 0.003,\,\varphi_{1} = 0.0075.$

**Figure 3:**
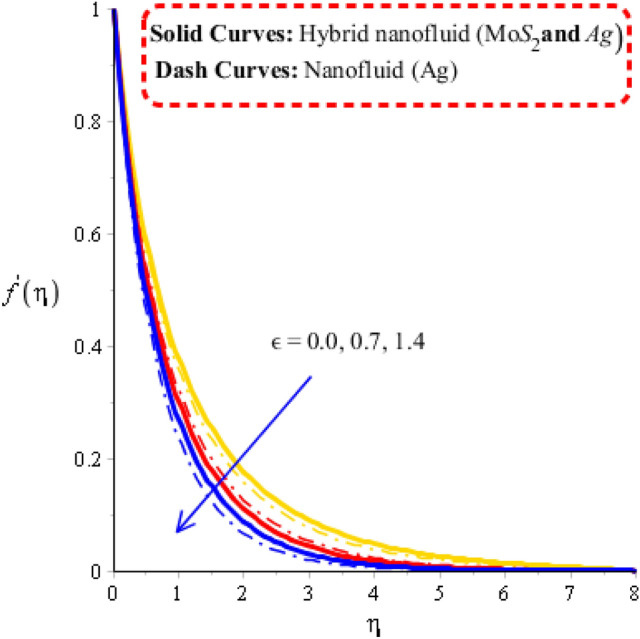
Velocity curves against $\varepsilon$ when $\,\lambda^{ * } = 1.3,\,\gamma = 0.7,\,Fr = 0.31,\,\Pr = 204,Hs = 1.5,\,\lambda = 1.3,\,\delta = 2.0,\,\varphi_{1} = 0.003,\,\varphi_{1} = 0.075.$

**Figure 4:**
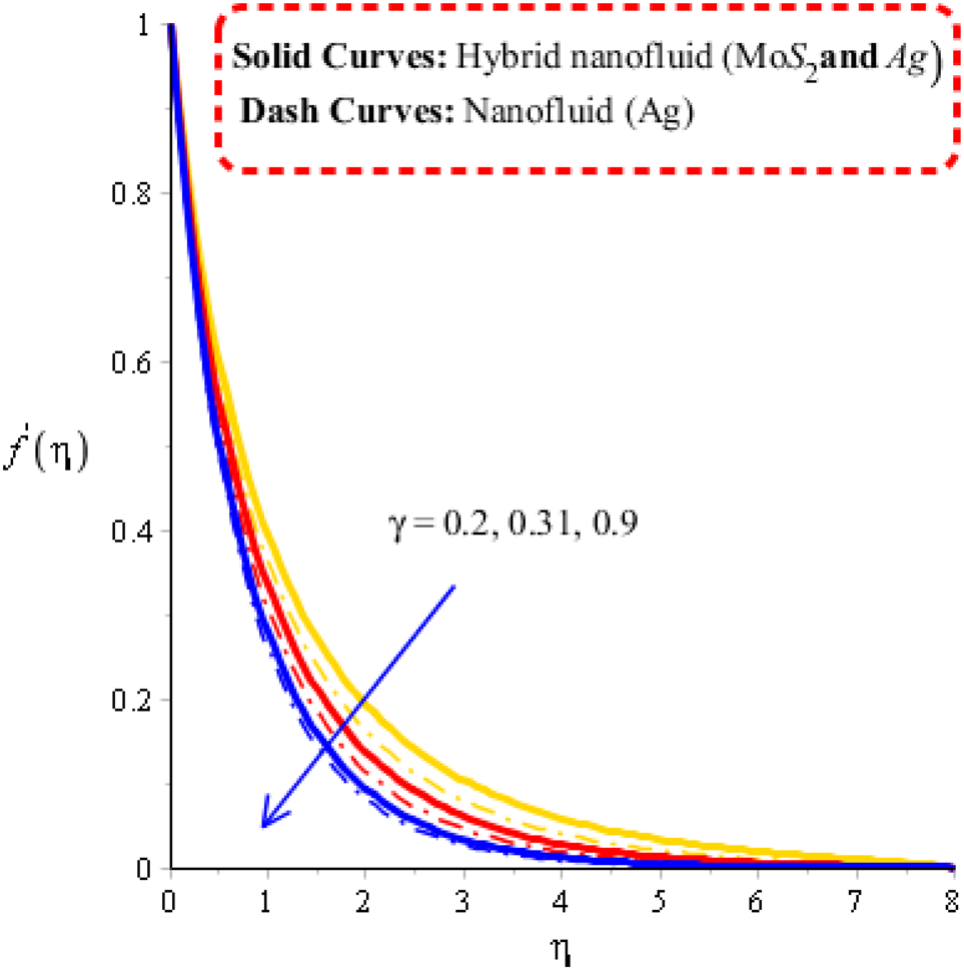
Velocity curves against $\gamma$ when $\varepsilon = 1.6,\,\lambda^{ * } = 1.2,\,Fr = 2.0,\,\Pr = 204,Hs = 0.7,\,\lambda = 1.4,\,\delta = 3.0,\,\varphi_{1} = 0.003,\,\varphi_{1} = 0.075.$

Measurement of velocity distribution is visualized versus change in Deborah number, Forchheimer number and porosity number. Related impacts of flow behaviors are observed by Figs. 2, 3 and 4. Distribution of fluid particles against the variation in Forchheimer number and porosity number is captured by Figs. 2 and 3. The concept of parameters related to $\varepsilon$ and $Fr$ is formulated in momentum equation due to Forchheimer porous effect on flow and resistive force (Darcy’s porous). It is noticed that velocity has linear relationship against Darcy’s while Forchheimer has nonlinear relation against flow. Phenomena of fluid particles experience resistive force during the flow for both cases of Forchheimer and Darcy’s porous. Hence, retardation force is investigated during flow generating by Forchheimer and Darcy’s porous. Moreover, layers related to MB (momentum boundary) are decreasing function versus variation in $\varepsilon$ and $Fr.$ Flow is generated by hybrid nanoparticles are higher than for the case of nanostructures. Figure 4 reflects the role Deborah number $\gamma$ on the velocity curves including hybrid nanomaterials and nano-structures. The motion of nanoparticles and hybrid nanomaterials is reduced versus the change in $\gamma$. This decrement behavior of flow is generated because of elastic nature (Maxwell fluid). Due to this nature of Maxwell liquid restores more deformation in view of flow analysis. MBLs (momentum boundary layers) are decreased due applying higher values of Deborah number. It is also investigated that approach related to hybrid nanoparticles are observed more efficient in view of maximum flow phenomena rather than approach related nanoparticles. Decreasing nature of flow characteristics is occurred due to elastic nature. It is also demonstrated that Maxwell liquid is more heated up as compared for the case of viscous fluid. Approach related to hybrid nanoparticles is observed more efficient to obtain maximum heat energy as compared for the case of approach related to nano-structures.

### Visualization of heat energy characteristics via change in physical parameters

**Figure 5:**
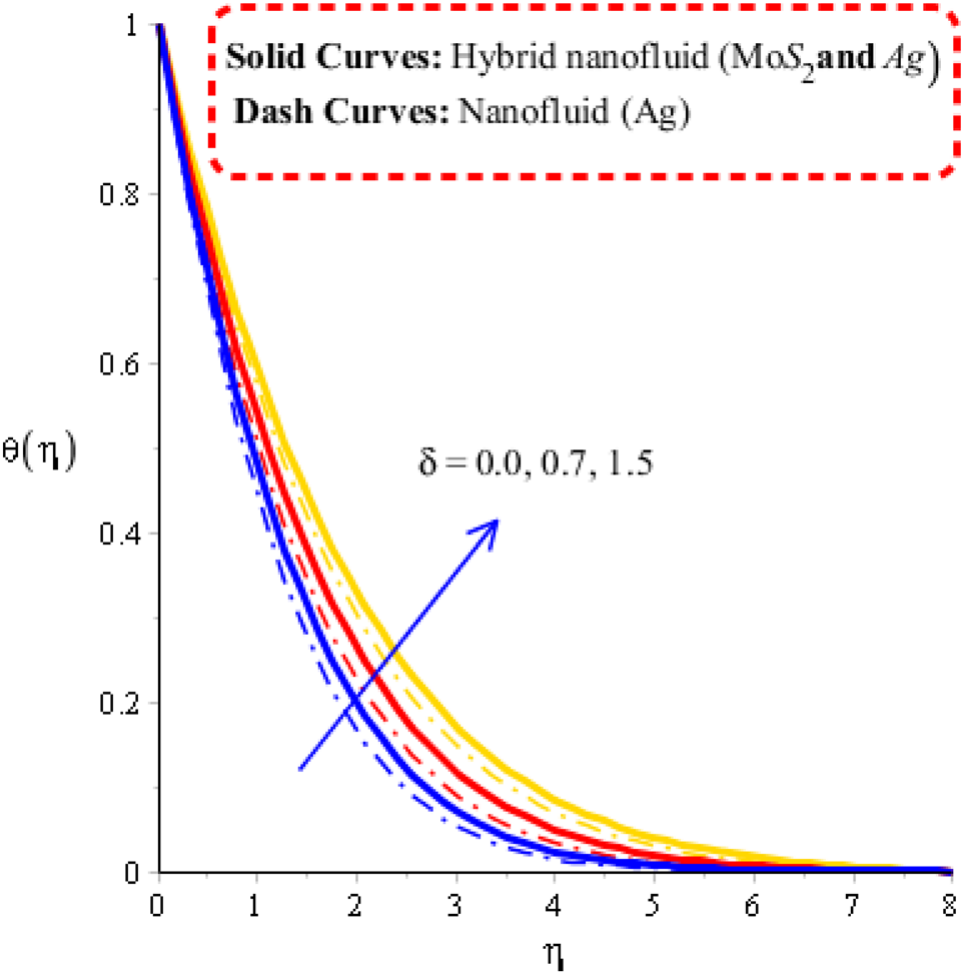
Temperature curves against $\delta$ when $\varepsilon = 0.3,\,\lambda^{ * } = 0.3,\,\gamma = 0.3,\,Fr = 1.6,\,\Pr = 204,Hs = - 1.3,\,\lambda = 1.3,\,\varphi_{1} = 0.003,\,\varphi_{1} = 0.075.$

**Figure 6:**
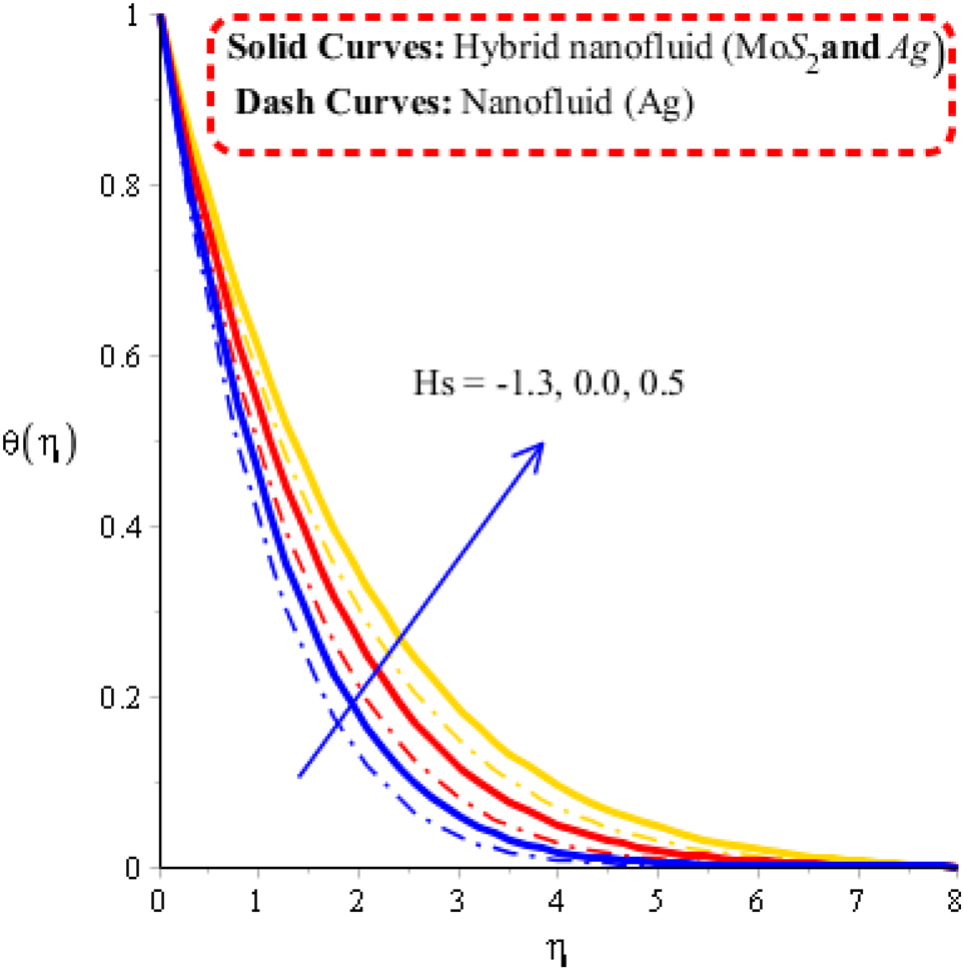
Temperature curves against $Hs$ when $\varepsilon = 1.5,\,\lambda^{ * } = 1.7,\,\gamma = 2.3,\,Fr = 0.5,\,\Pr = 204,\,\lambda = 2.1,\,\delta = 1.2,\,\varphi_{1} = 0.003,\,\varphi_{1} = 0.075.$

**Figure 7:**
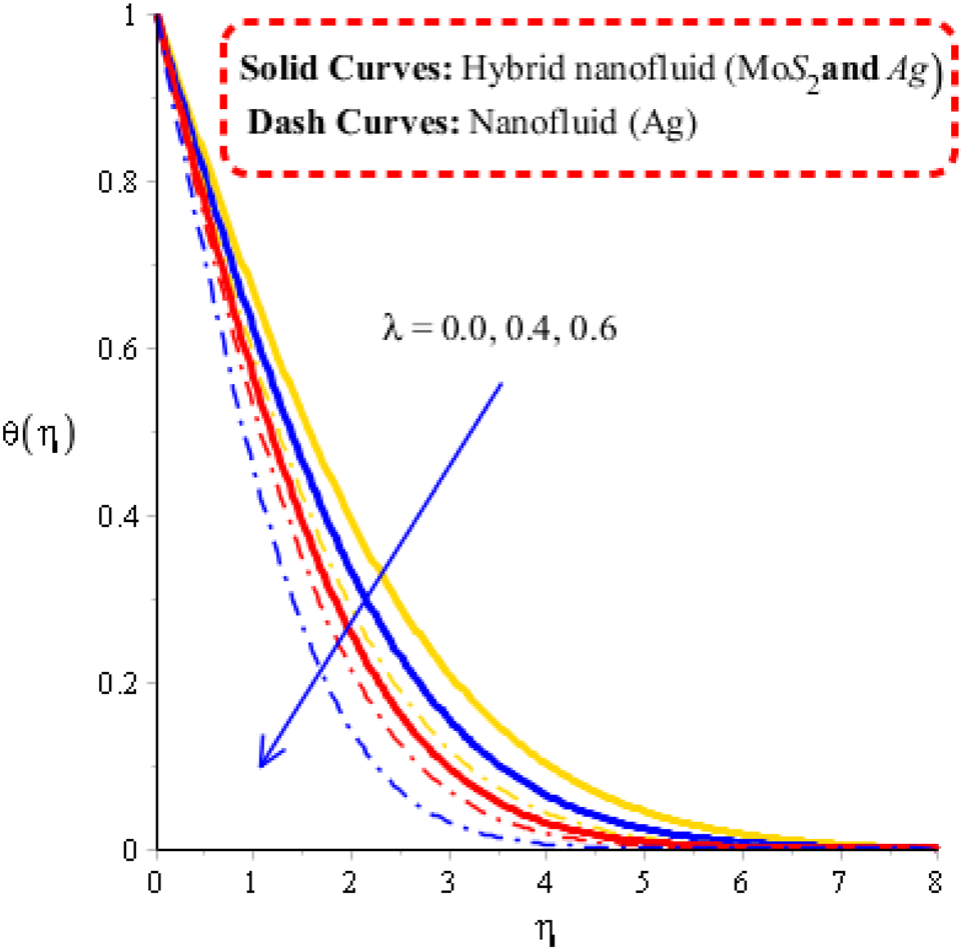
Temperature curves against $\lambda$ when $\varepsilon = 0.2,\,\lambda^{ * } = 0.4,\,\gamma = 2.3,\,Fr = 0.5,\,\Pr = 204,Hs = 2.3,\,\,\delta = 3.0,\varphi_{1} = 0.003,\,\varphi_{1} = 0.075.$

**Figure 8:**
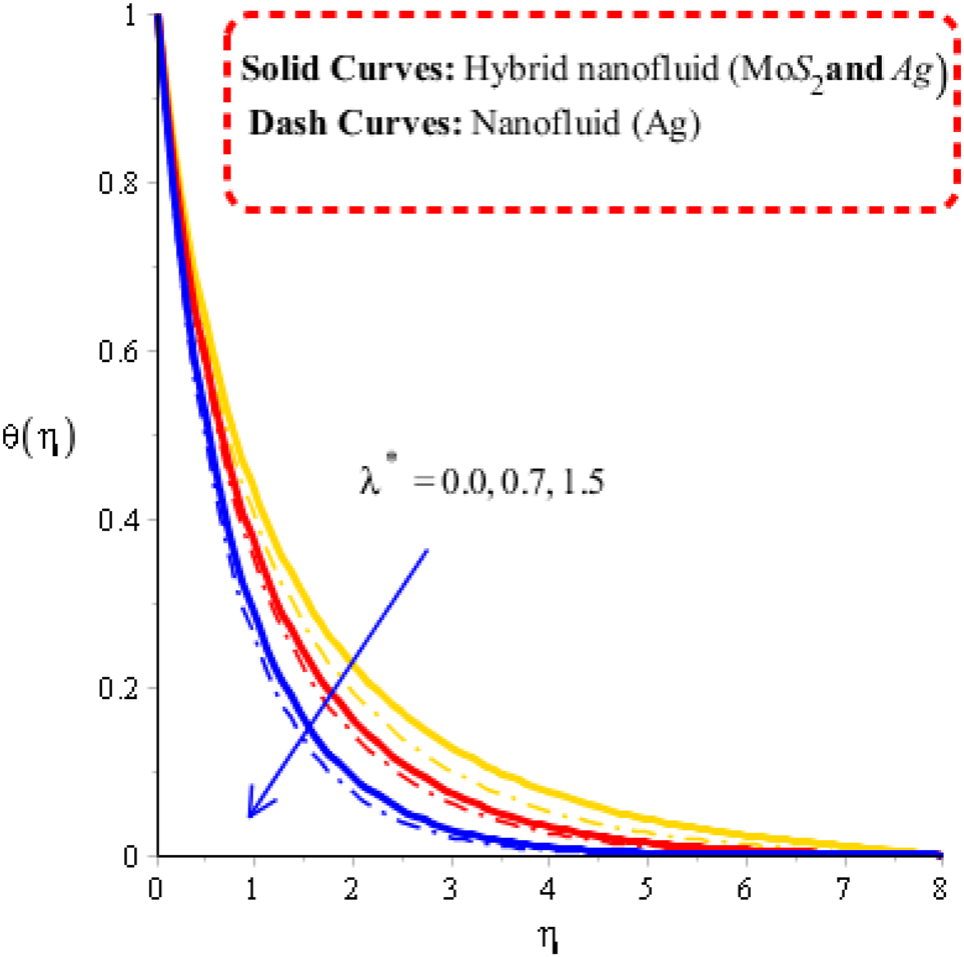
Temperature curves against $\lambda^{ * }$ when $\varepsilon = 0.2,\,\,\gamma = 0.7,\,Fr = 0.26,\,\Pr = 204,Hs = - 1.7,\,\lambda = 1.3,\,\delta = 0.53,\,\varphi_{1} = 0.003,\,\varphi_{1} = 0.075.$

The parameters called heat generation ($Hs$), Deborah number ($\gamma$), unsteadiness ($\lambda^{ * }$) and time relaxation ($\delta$) are tested on temperature profile including approach of hybrid nanoparticles. The comparative simulations among nanoparticles and hybrid nano-structures are visualized by Figs. 5, 6, 7 and 8. The variation of heat energy against Deborah number is taken out by Fig. 5. Heat energy increases when time relaxation number is enhanced. The term related to relaxation time is arisen in dimensionless energy equation using concept of non-Fourier’s law. From Fig. 5, it is noticed that heat energy due to Fourier’s law of heat conduction is less than heat energy for the case of concept of non-Fourier’s law. An increment in heat energy is noticed when time relaxation number is increased. Figure 6 is plotted for measurement of heat energy with respect to large values of heat generation number. In this figure, positive values indicate heat generation while negative values reveal demonstrates heat absorption mechanism. Moreover, heating and cooling phenomena are indicated by positive and negative value, respectively. Maximum heat generation is made the reason to obtain maximum heat energy. External heat source is occurred due heat generation number. Thickness related to (MB) is inclined using higher values of heat generation number. A role of $\lambda$ on temperature curves is estimated by Fig. 7. The concept of $\lambda$ is formulate due to temperature difference in momentum equations. Temperature curves are enhanced using higher values of $\lambda$. Temperature curves for the case nano-structures are reduced temperature curves for hybrid nanoparticles. Hence, $\lambda$ is investigated as a suitable parameter to generate more heat energy. Figure 8 illustrates the impact of unsteadiness parameter on heat energy profile including approach of hybrid nanoparticles. The unsteady flow is occurred due to unsteadiness parameter. Mathematically, it is the ratio of constant parameter (due to time) and parameter (due to stretching of wall). Flow becomes slow down by increasing the values of unsteadiness parameter because of inverse relation. Fluid is heated up applying large values of unsteadiness parameter. In comparative point of view, hybrid nanoparticles are observed more efficient to obtain higher amount for heat energy.

### Behavior of temperature gradient and surface force

Table 3 is prepared to study the impact of Prandtl number on heat transfer rate. From the table, it is clear that rate of heat transfer enhances against Prandtl number. Furthermore, it is recorded that the obtained data is an excellent settlement with the studies reported in Ref.^39^. Distribution in temperature gradient (Nusselt number) and surface force (skin friction coefficient) is observed versus change in $$\gamma ,$$ $$Fr,$$$$Hs$$ and $$\delta$$. These impacts are simulated by Table 4. It is observed that reduction is found in temperature gradient using large values of $$\gamma ,$$ $$Hs$$ and $$\delta$$. Moreover, rate of heat transfer for the case of hybrid nanoparticles is higher than rate of heat transfers for nanofluid. Surface force increases versus the large values of $$\gamma ,$$ $$Hs$$ and $$Fr.$$ Further, approach related to hybrid nanoparticles are observed as significant in view of surface force as compared for the case of nanofluid.

**Table 3:** Validation of results regarding temperature gradient when $\varepsilon = 0.35,\,\lambda^{ * } = 0,\,\gamma = 0,\,Fr = 0.5,\,Hs = 0,\,\lambda = 0,\,\delta = 0,\,\varphi_{1} = \varphi_{2} = 0.$

	Bilal et al.^39^	Present results
**Pr**		
0.20	0.1693	0.1688371
0.70	0.4540	0.4541030
2.00	0.9114	0.9113893

**Table 4:** Numerical values of − $$(Re)^{1/2} Nu$$ and − $$(Re)^{1/2} C_{f}$$ versus $$\gamma ,$$$$Fr,$$$$Hs$$ and $$\delta$$^21^ when $$\varepsilon = 0.3,\,\lambda^{ * } = 0.7,\,\,\Pr = 204,\,\lambda = 1.3,$$ $$\,\varphi_{1} = 0.003,\,\varphi_{1} = 0.075.$$

		Nanoparticles					Hybrid nanoparticles		
		$(Re)^{1/2} C_{f}$	Error	$(Re)^{1/2} Nu$	Error	$(Re)^{1/2} C_{f}$	Error	$(Re)^{1/2} Nu$	Error
$\gamma$	0.0	0.68347	$6 \times 10^{ - 8}$	0.20378	$7 \times 10^{ - 8}$	5.1854	$5 \times 10^{ - 8}$	3.50901	$7 \times 10^{ - 8}$
	0.4	0.78369	$6 \times 10^{ - 8}$	0.20132	$6.6 \times 10^{ - 8}$	5.2147	$4.7 \times 10^{ - 8}$	3.30984	$6.04 \times 10^{ - 8}$
	0.8	0.79257	$5.7 \times 10^{ - 8}$	0.10463	$5.2 \times 10^{ - 8}$	5.5147	$4.1 \times 10^{ - 8}$	3.21069	$5.31 \times 10^{ - 8}$
$Fr$	0.0	0.74058	$5 \times 10^{ - 8}$	0.14246	$5.1 \times 10^{ - 8}$	5.3441	$3.7 \times 10^{ - 8}$	3.14617	$4.3 \times 10^{ - 8}$
	0.7	0.77769	$4.6 \times 10^{ - 8}$	0.14438	$3.9 \times 10^{ - 8}$	5.5152	$3.1 \times 10^{ - 8}$	3.14914	$3 \times 10^{ - 8}$
	1.3	0.79600	$4 \times 10^{ - 8}$	0.14680	$2.3 \times 10^{ - 8}$	5.8441	$2.5 \times 10^{ - 8}$	3.15157	$2.73 \times 10^{ - 8}$
$Hs$	− 1.3	0.70793	$3.7 \times 10^{ - 8}$	0.55061	$2 \times 10^{ - 8}$	2.3238	$2.2 \times 10^{ - 8}$	3.42032	$2 \times 10^{ - 8}$
	0.3	0.39701	$3.3 \times 10^{ - 8}$	0.33040	$1.8 \times 10^{ - 8}$	2.2411	$1.7 \times 10^{ - 8}$	3.20134	$1.74 \times 10^{ - 8}$
	0.7	0.19601	$3 \times 10^{ - 9}$	0.14039	$1.2 \times 10^{ - 8}$	2.1953	$1 \times 10^{ - 8}$	3.11143	$1.1 \times 10^{ - 8}$
$\delta$	0.0	0.08757	$2.7 \times 10^{ - 9}$	0.17348	$1 \times 10^{ - 8}$	2.4216	$0.8 \times 10^{ - 9}$	3.17887	$0.93 \times 10^{ - 9}$
	0.2	0.08212	$1 \times 10^{ - 9}$	0.20652	$0.91 \times 10^{ - 9}$	2.2149	$0.6 \times 10^{ - 9}$	3.01252	$0.5 \times 10^{ - 9}$
	0.5	0.08110	$0.9 \times 10^{ - 9}$	0.14790	$0.09 \times 10^{ - 8}$	2.3841	$0.2 \times 10^{ - 9}$	3.00199	$0.23 \times 10^{ - 9}$

## Consequences of current problem

Characteristics of heat energy in Maxwell fluid suspending by hybrid nanoparticles pas melting vertical surface are modeled. Theories related to non-Fourier’s law and Darcy’s Forchheimer law is visualized in heat transfer phenomena along with heat generation. Finite element method is used to know results of current study. The main findings are listed below:

- Thermal growth is enhanced for the case of hybrid nano-structures rather than for case of nanofluid.
- Maximum thermal energy is achieved when heat generation number is increased.
- Convergence analysis of considered problem is confirmed up to 300 elements.
- Heat energy and TBL (thermal boundary layer) are augmented against large values of unsteadiness number and relation time parameter.
- Motion into hybrid nanoparticles and nanoparticles becomes slow down versus higher values of Forchheimer and Darcy’s porous numbers.
- Thickness of MBL (momentum boundary layer) is dominated for the case of hybrid nanoparticles rather than case of nanoparticles.
- Minimum temperature gradient is measured versus variation in time relaxation and fluid number while skin friction coefficient is increased against variation heat generation and Forchheimer numbers.

## Data Availability

The data used to support this study are included in the Manuscript.
